# Keratan sulfate, an electrosensory neurosentient bioresponsive cell instructive glycosaminoglycan

**DOI:** 10.1093/glycob/cwae014

**Published:** 2024-02-20

**Authors:** James Melrose

**Affiliations:** Graduate School of Biomedical Engineering, University of New South Wales, Sydney, NSW 2052, Australia; Raymond Purves Laboratory, Institute of Bone and Joint Research, Kolling Institute of Medical Research, Northern Sydney Local Health District, Royal North Shore Hospital, St. Leonards, NSW 2065, Australia; Sydney Medical School, Northern, University of Sydney at Royal North Shore Hospital, St. Leonards, NSW 2065, Australia

**Keywords:** cochlea, electrolocation, electro-mechanotransduction, keratan sulfate, neurosensory processes

## Abstract

The roles of keratan sulfate (KS) as a proton detection glycosaminoglycan in neurosensory processes in the central and peripheral nervous systems is reviewed. The functional properties of the KS-proteoglycans aggrecan, phosphacan, podocalyxcin as components of perineuronal nets in neurosensory processes in neuronal plasticity, cognitive learning and memory are also discussed. KS-glycoconjugate neurosensory gels used in electrolocation in elasmobranch fish species and KS substituted mucin like conjugates in some tissue contexts in mammals need to be considered in sensory signalling. Parallels are drawn between KS’s roles in elasmobranch fish neurosensory processes and its roles in mammalian electro mechanical transduction of acoustic liquid displacement signals in the cochlea by the tectorial membrane and stereocilia of sensory inner and outer hair cells into neural signals for sound interpretation. The sophisticated structural and functional proteins which maintain the unique high precision physical properties of stereocilia in the detection, transmittance and interpretation of acoustic signals in the hearing process are important. The maintenance of the material properties of stereocilia are essential in sound transmission processes. Specific, emerging roles for low sulfation KS in sensory bioregulation are contrasted with the properties of high charge density KS isoforms. Some speculations are made on how the molecular and electrical properties of KS may be of potential application in futuristic nanoelectronic, memristor technology in advanced ultrafast computing devices with low energy requirements in nanomachines, nanobots or molecular switches which could be potentially useful in artificial synapse development. Application of KS in such innovative areas in bioregulation are eagerly awaited.

## Graphical Abstract

**Graphical Abstract:**
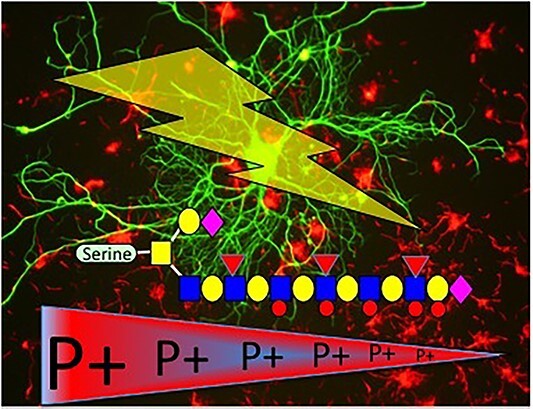

## Introduction

Keratan sulphate (KS) is a complex, neurosentient, electrosensory multifunctional glycosaminoglycan (GAG) which has unique functional capabilities (Caterson and Melrose 2018; Hayes and Melrose 2018; Melrose 2019b). A recent proteomic study on corneal KS (KSI) using a microarray of 8,268 proteins and customized array of 85 extracellular nerve growth factor protein epitopes uncovered a wealth of data pointing to potential roles for KS in neuronal cell-signaling (Conrad et al. 2010). Highly sulfated KS interacts with 217 microarray proteins including 75 kinases, membrane, secreted and cytoskeletal proteins and a number of nerve regulatory proteins. Interactions of KS with Robo-Slit resulted in downstream activation of Rho GTPases regulating intracellular cell signaling actin polymerization dynamics, cytoskeletal re-organization, cell signaling and effects on cellular migration, cell shape and cellular proliferation (Melrose 2018). Short range inhibitory signals delivered by semaphorin-plexin and neuropilin receptor interactions regulate Rho GTPases critical to axonal guidance in neural development and repair. The ability to perceive ion-fluxes provided by KS facilitates the sensory capability of cells in the perception and responses to dynamic environmental biomechanical change and has even been proposed as a mechanism whereby long term potentiation of memory occurs (Eccles 1983). In neurons, the ability to sense and control ion-fluxes is highly advanced generating action potentials which are the basis of synaptic function (Camire and Topolnik 2014). Furthermore, KS and its associated Ca2+ counterions may act as a calcium reservoir in egg shell production (Ha et al. 2007; Du et al. 2015) and mineralization of bone with several KS-SLRPs proposed to have roles in bone formation (Kinne and Fisher 1987; Sommarin et al. 1998; Wendel et al. 1998; Gori et al. 2001; Nakamura et al. 2001; Igwe et al. 2011; Nikdin et al. 2012).

## KS and proton gradients

Proton gradients have been proposed to be an emotive life force whose origins can be traced back to the initiation of life. An awareness that the transport of positively charged protons along a pH gradient served to generate energy in cellular systems established membrane energetics as a central life force science (Lane and Martin 2012; Lane 2017). The proton gradient is a form of potential energy that is used by mitochondria and the ATP synthase complex to produce ATP, a fundamental energy molecule upon which all eukaryotic life relies for aerobic glycolysis and energy production (Alberts et al. 2002). Furthermore, oxidative phosphorylation by mitochondria is central to phosphorylation/dephosporylation biosynthetic events forming metabolites that regulate cell signaling in all mammals providing physiological control of cellular metabolism. Rather than just being considered purely as the powerhouse of the cell, the mitochondrion is now appreciated to have further roles that contribute to cellular and organismal health (Monzel et al. 2023). Mitochondria transduce metabolic, biochemical, neuroendocrine, and other local or systemic signals to facilitate organismal adaptation. Mitochondrial signal transduction now establishes specific communication roles in mitochondrial biology (Picard and Shirihai 2022). The mitochondrion is thus much more than just an energy production system, and protein biosynthetic factory. Proton gradients are key drivers of these life sustaining processes.

Proton (H^+^) conductivity is important in many natural cellular processes (DeCoursey and Cherny 2000) including oxidative phosphorylation in mitochondria and energy production uncoupling of membrane potentials during membrane polarization and neural activation (Mitchell 1966) and in the priming of cells for proliferation, apoptosis or migration (Ye and Steiger 2015; Love et al. 2018), controlling cell and tissue polarity and cell regulation (Valero et al. 2008). An analysis of the proton conductivity of GAGs shows KS is the best proton detector generating hydroxonium or hydronium ions through co-operative inter and intramolecular hydrogen bonding with the detected proton (Chang and Minc 2014). The conduction of protons occurs through hydrogen bond interactions between water and hydrophilic residues on the KS chain (Deplazes et al. 2017). In Nature, protons mediate metabolic processes through enzymatic reactions. A KS substituted-mucin-like glycoconjugate gel isolated from the Ampullae of Lorenzini, a sensory skin pore system in elasmobranch fish (sharks, rays, skates), is the best proton detection polymer known in Nature (Josberger et al. 2016; Selberg et al. 2019). Such KS-mucinous deposits signal through neurosensory networks to detect electric fields generated by the muscular activity of preyfish species in a process known as electrolocation (Zhang et al. 2018). KS is the most sensitive proton detection molecule known in nature (Josberger et al. 2016).

## The sulfation status of KS is an important functional determinant

Sulfation of KS is an important functional determinant that determines the antigenicity of KS species used as immunogens. Thus it was not surprising that when KS monoclonal antibodies (MAbs) were first developed such as 5D4 (Caterson et al. 1983) and MZ15 (Mehmet et al. 1986; Craig et al. 1987) these were to highly sulphated regions on KS. With their availability as analytical tools it was inevitable that studies with these antibodies would historically dominate examination of the roles of KS in tissue development and pathology. However KSI also contains monosulphated regions and stretches of non-sulphated polylactosamine but their contributions to the biology of KS were overshadowed by studies using MAbs 5D4 and MZ15. With the development of MAbs (1B4, R10G) (Kerr 2005; Young et al. 2007b) to low sulphated regions of KS it has now become possible to examine the roles of these low sulphated regions on KS biology. Furthermore, a number of proteoglycans bearing low sulfation KS chains have now been identified and the potential roles of low sulfation KS has become an experimental possibility.

## The responses elicited by high and low sulfation KS

**Fig. 1:**
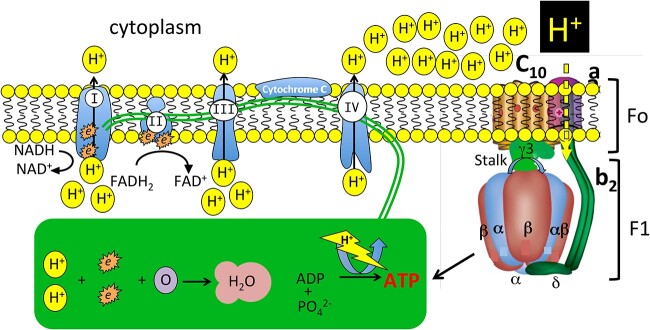
Schematic showing how the mitochondrial electron transport chain drives oxidative phosphorylation and ATP synthesis by ATP synthase. Complex I in the electron transfer chain strips protons (H) and electrons (*e*) from NADH and these are pumped through the inner mitochondrial membrane, transferred to coenzyme Q (CoQ) and then to complex III. Complex II also receives *e* and H from FADH2 via the citric acid cycle and transfers these via CoQ to complex III which pumps large amounts of H across the inner mitochondrial membrane. Cytochrome C transfers *e* to complex IV then to an oxygen molecule where binding to H forms water. Build up of H on the cytoplasmic side of the inner mitochondrial membrane drives ATP production from ADP by ATP synthase by a process known as oxidative phosphorylation. F1Fo-ATP synthase is a membrane protein complex that converts a cell’s transmembrane proton gradient into chemical energy which is stored as ATP. ATP synthase consists of two molecular motors, Fo and F1, that are coupled by a central stalk. The membrane unit, Fo, converts the transmembrane electrochemical potential into mechanical rotation of a rotor in Fo and the physically connected central stalk drives the synthesis of ATP by the F1 α, β, δ subunits. Figure modified from (Aksimentiev et al. 2004).

Historically, the biological response shown to be elicited by GAGs was normally undertaken in studies which examined a high charge density GAG and this was the major focus of GAG pathobiology. Sulfation density and spatial conformational presentations of GAGs strongly correlated with their functional properties and were recognized as strong functional determinants facilitating strong interactions with many signaling molecules that controlled cellular behaviour and regulated physiological processes. Few studies at this time assessed whether low sulfation KS had interactive properties of interest. Analytical reagents which can detect KS of lower sulfation density only became available fairly recently and it is now possible to identify some specific proteoglycan isoforms containing low charge density KS chains and roles for these in specific cellular and tissue contexts are now emerging. Innovative work on the sensory gel of elasmobranch fish species uncovered some interesting findings with low sulfation KS and showed it has ultrasensitive proton detection capability and is the most sensitive proton detection system so far known in nature. Given the fundamental roles proton gradients play in essential life processes this illustrates the importance of low sulfation KS as a bioregulatory system. Proton gradients are not only of importance in eukaryotic systems but in all forms of life. In eukaryotes mitochondria use the energy in the proton electrochemical gradient to make ATP, a fundamental energy source in life processes (Fig. 1). Proton gradients created by the photosynthetic process provide the energy necessary for ATP production. Furthermore, proton gradients are also of interest in nanoelectronic systems in evolving futuristic studies aimed at developing electronic molecular switches not only in biological systems to control physiological processes (synaptic processing) but also in microelectronic devices in digital and nanoelectronics such as memristors for use in high speed, low energy computation and data storage (Nichols and Higgins 2015; Bueno and Davis 2020; Duan et al. 2024; Xia et al. 2024), and in nano-bots (Aydin et al. 2019).

Many authors have written about the information storage and transfer properties of GAGs and their roles in cellular regulation (Vallet et al. 2022; Schwartz and Domowicz 2023) however it is the regulation of proton transfer which could be considered the operating system for information encoded by the GAG fine structure and in this respect this could be considered a biological equivalent of digital information transfer technology which has been applied in computing systems and also explains why KS is particularly relevant in neural processes. The neuron is particularly sensitive to electrical stimulation and this is essential for neural activation and neurotransduction (Melrose 2018; Hayes and Melrose 2018; Melrose 2019a; Perez et al. 2023).

## The electrosensory gel of the ampoules of Lorenzini

**Fig. 2:**
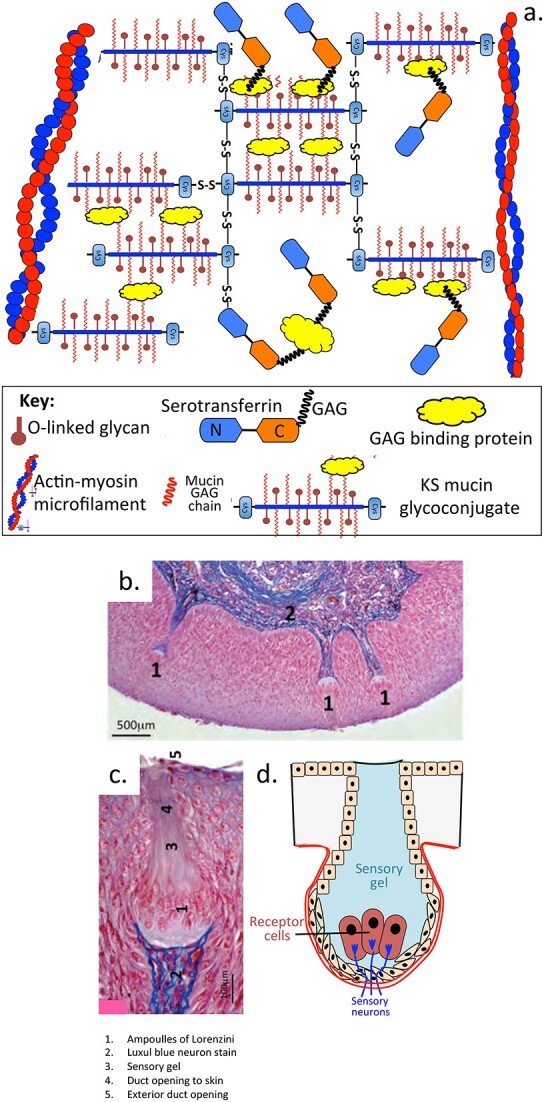
A schematic of the major components of the ultrasensitive proton detecting KS glycoconjugate identified in the sensory skin-pores (Ampoulle of Lorenzini) of elasmobranch fish species as proposed by Zhang et al. 2018 (Zhang et al. 2018) (a). Luxol blue/H & E histology of a section of shark skin with a sensory pore filled with KS glycoconjugates showing this interacting with the sensory nerves interfacing with the ampoule (b, c, d). Luxol blue stains myelin and visualizes myelin/myelinated axons and Nissl bodies in nerves. Segments b, c reproduced from (Melrose 2019a).

The ampullae (a Roman term for a distinctly shaped bulbous wine cask) of Lorenzini were first identified as an electrosensory system in a network of jelly-filled pores located on the snout and head of elasmobranch fish species (sharks, skates, and rays) by Marcello Malpighi and a detailed description was later provided by the Italian scientist Stefano Lorenzini in 1678, whom these structures are named after (Fig. 2). Early descriptions of this electrosensory system are also provided by several other authors (Kalmijn 1971; Fishelson and Baranes 1999). However it was only relatively recently that the identity of the electrosensory gel found in these pores was identified and its molecular properties determined (Josberger et al. 2016; Zhang et al. 2018; Selberg et al. 2019; Phillips et al. 2021). This electrosensory gel contains KS, and is one the purest sources of this glycosaminoglycan, KS occurs as a low sulfation isoform attached to a mucin-like conjugate (Zhang et al. 2018). This conjugate is stabilized by disulphide bonding and actin microfibrils. Serotransferrin, itself a protein which can be substituted with KS (Magro et al. 2003) is also a component of this complex as is another unidentified GAG binding protein. Subsequent studies showed this electrosensory gel was an ultrasensitive proton binding complex and the most sensitive proton detection system known in nature (Phillips et al. 2021). KS has superior proton binding properties than all other GAGs (Selberg et al. 2019). The gel contained in the Ampullae has electrosensory properties and is used by elasmobranch fish to electrolocate prey fish through the protons they emit during muscular exertion.

During evolution electroreception was lost as a sense when amphibian species transitioned to a terrestrial lifestyle. The present day salamanders and gymnophians have retained electroreceptors (Northcutt et al. 1995). Gymnophians are members of the Caecilian family of limbless, serpentine amphibians with small or nonexistent eyes which live mostly hidden in soil or under gravel in streambeds making these uncommonly encountered species.

**Fig. 3:**
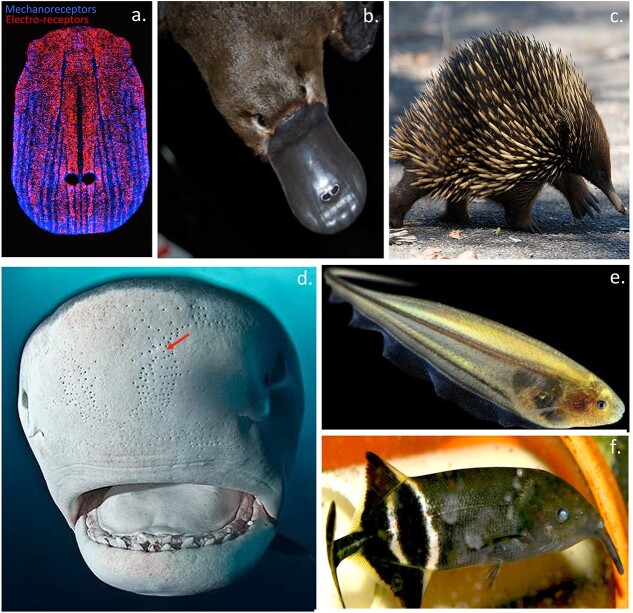
Some examples of terrestrial animals and fish that utilise electrolocation as a sense to map their environment or to hunt for food items. Electroreceptors and mechanoreceptors in the duck-billed platypus (a, b). Echidna also displays electrolocation (c). Elasmobranch fish (sharks and rays) have electrosensory pores (arrows) filled with the electrosensory gel that facilitates electrolocation (d). Knife fish (e) and elephant fish (f) also utilise electrolocation to assess their aqueous environment. Image modified from (Melrose 2019a).

Two terrestrial monotreme animals, the Duck-billed platypus (Fig. 3a and b) and Echidna (Fig. 3c) are the only terrestrial animals known to have retained electrolocation as a sense which they use to search for food species (Melrose 2019a). The Duck billed platypus has modified trigeminal nerve fibres which are sensitive enough to detect weak electric fields by electrolocation. The bill of the platypus also contains a large number of mechanoreceptors which along with the bill electroreceptors are used to hunt for food species. The platypus is a nocturnal feeder and does so with its eyes and nostrils firmly shut thus electrolocation is very important to the platypus in the search for food items. Active electrolocation is used by sharks and rays to locate prey through sensory pores in their nose region (Fig. 3d). Electrolocation is also used by two groups of weakly electric fish, the *Gymnotiformes* (knifefishes) (Fig. 3e) and the *Mormyridae* (elephant fishes) (Fig. 3f). These fish generate an electric field emitted from an electric organ located in modified tail muscles to surround themselves in a weak electric field which they use to assess their environment. These fish use electrolocation for social interaction allowing them to sense the sexual maturity of potential mating parners and to sense their status within the hierarchy of a given fish group (Melrose 2019a). Dolphins and whales also emit controlled sound pulses (echolocation) generated by organs in their head regions to analyse their environment and for social communication by detecting the transmitted echo they receive back from disturbances in this emitted sound field (Czech-Damal et al. 2012; Czech-Damal et al. 2013; Hüttner et al. 2023). Water is a relatively good electrical conductor, and many aquatic and amphibious animals—including sharks, rays, catfish, and salamanders—are able to sense and respond to naturally occurring electric fields in order to detect food species and predatory threats. Duck-billed platypuses and the echidna also display electroreception, which is used underwater or in moist environments to search for food items.

Some terrestrial arthropods can also detect weak electrical fields through sensory hairs attached to their exo-skeletons using aero-acoustic sensing (Palmer et al. 2022; Palmer et al. 2023). Thread-like hairs that cover a honey or bumblebee sense weak electric fields in the absence of a conducting medium (Sutton et al. 2016). Honey bees have no ears but vibrations from these hairs are directly connected to the bee exoskeleton and feed acoustic signals directly into their neural systems. Aerial electrolocation in bees aids in nectar collection from the most suitable flower heads and allows the bee to discriminate between rewarding and unrewarding flowerheads. Electrostatic pollen transfer to the bee also occurs during nectar collection which is ecologically important in plant pollination (Lihoreau and Raine 2013). Insect pollinators detect and select flowers by their colours, shapes, patterns and fragrant volatiles (Raguso 2008; Clarke et al. 2013; Rader et al. 2016). Electrolocation is a further highly efficient selection modality which allows bees to detect the weak electric fields around flowers and also allows bees to communicate the geographic location of these productive flower groups to other members of the bee colony (Clarke et al. 2017). While bees carry out fewer total flower visits than other pollinators, they are responsible for about half of all crop pollinations (Rader et al. 2016).

## KS heterogeneity

**Fig. 4:**
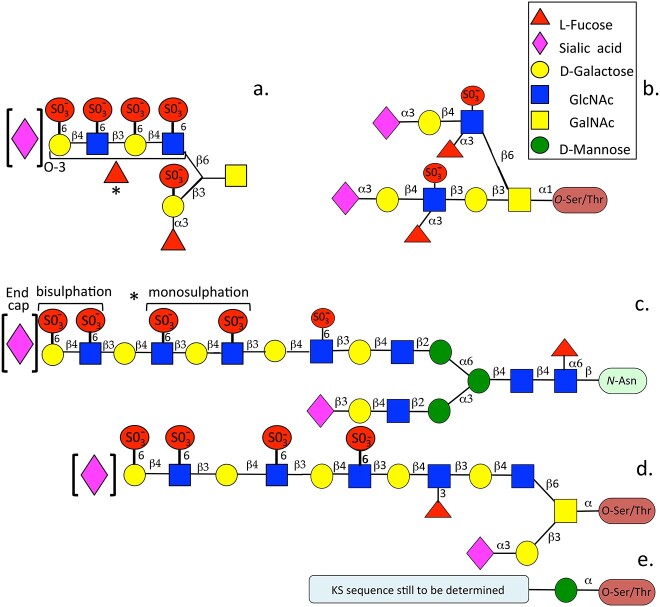
Schematic depiction of typical glycan organization in the GAGs associated with mucins (a, b), N-linked corneal type I KS (c), O- linked skeletal KSII (d) and O-linked cerebral type III KS (e) showing sulfation positions, fucosylation and sialic acid end-capping. Image modified from (Caterson and Melrose 2018).

Keratan sulphate (KS) is composed of the repeat disaccharide D-galactose β1 → 4 glycosidically linked to N-acetyl glucosamine. A family of glycosyl transferase and sulphotransferases sequentially add D-galactose (D-Gal) then N-acetyl glucosamine (GlcNAc) to C6 of the KS acceptor oligosaccharide (Funderburgh 2000; Funderburgh 2002). KS is the only branched GAG however the C3 arm of the linkage oligosaccharide undergoes premature truncation and is often capped in sialic acid. D-Gal and GlcNAc on the nascent KS chain can both undergo sulfation at C6, this occurs more frequently on GlcNAc than D-Gal. Sulfation along the developing KS chain is not uniform but occurs at disulphated regions involving GlcNAc and D-Gal and monosulphated (GlcNAc only) regions of variable length (Caterson and Melrose 2018). D-Gal sulphotransferase acts once GlcNAc is sulphated to give rise to a disulphated KS disaccharide however in KS D-Gal is less frequently sulphated leading to heterogeneous distributions of mono- or disulphated regions while non-sulphated lactosamine regions can also occur along a given KS chain towards the linkage oligosaccharide region. Variably sulphated regions in KS define its interactive properties with growth factors, morphogens and cytokines and determine the functional properties of KS in tissues (Weyers et al. 2013). KS has three different linkage structures to proteoglycan core proteins. These include Asparagine (N-linked), Threonine or Serine residues (O-linked) or Mannose residues (O-linked) which are categorised into KS I-III, L-fucose and sialic acid groups may also be randomly added to KS chains at C3 along the KS chain or as capping structures respectively at the non-reducing terminus. The chain size distribution and degree of sulfation of KS chains increases with tissue maturation and its pathological status. KS is a heterogeneous GAG and exhibits both variation in chain length and in sulfation along the KS chain leading to a considerable level of size and charge heterogeneity. Tethered and soluble mucins can also be modified with KS chains and in this respect these resemble the mucin-like KS glycoconjugate previously discussed which has been identified in the sensory pores of elasmobranch fish species (Kesimer et al. 2012; Carpenter and Kesimer 2021) (Fig. 4).

## Highly sulphated KS is a biomarker of tumours

High charge density KS is associated with a number of tumours and a diagnostic marker but it may also arise in tissues subjected to trauma. Highly sulphated KS is prominent in carcinomas of the genital tract (Miyamoto et al. 2011), prostatic secretory cells (Cohen et al. 2000), brain and ovarian tumours (Whitham et al. 1999), papillary carcinomas of the human thyroid gland (Ito et al. 1996) and granular cell tumours (Ehara and Katsuyama 1990), malignant astrocytic tumours (Kato et al. 2008; Hayatsu et al. 2008a), and glioblastoma (Hayatsu et al. 2008b). The human embryonic carcinoma marker antigen TRA-1-60 has been identified as a sialylated KS proteoglycan (Schopperle and DeWolf 2007).

## KS substituted mucins

Tethered and secreted mucins contain GalNAc, GlcNAc, Gal, Fuc, N-acetyl neuraminic acid attached to their core proteins through *O*-linkage to Ser and Thr residues on their tandem repeat domains leading to a bottle brush type structure reminiscent of PGs (Aplin et al. 1998; Kesimer et al. 2012). The GlcNAc residues of members of the mucin glycoprotein family can also act as acceptor molecules for the addition of D-Gal and GlcNAc residues by a family of sulphotransferases in some tissue contexts and these can be sulphated to variable degree leading to mucin KS glycoforms (Brockhausen 2003). Cultured human tracheobronchial epithelial cells synthesise 5D4 KS positive MUC1, MUC4, and MUC16 tethered to cilia and microcilia in the epithelial glycocalyx (Kesimer et al. 2012). MUC-1 in human endometrial tissue carries 5D4 positive KS and a sialo-KS epitope is recognized by Mab D9B1 (Aplin et al. 1998). With the recent development of antibodies which identify low sulfation motifs on KS, proteoglycans and KS-glycoconjugates devoid of the multisulphated regions have been identified and roles for these KS isoforms are slowly emerging.

## Podocalyxcin, a KS sialoglycoprotein

Human embryonic pluripotent stem cells, express the antigens TRA-1-60 and TRA-1-81 antigens which are located on the cell adhesion KS-proteoglycan podocalyxcin (Schopperle and DeWolf 2007). Podocalyxcin is a 240 kDa anti-adhesive, mucin-like transmembrane cell surface CS and KS modified sialoproteoglycan implicated in the development of aggressive forms of cancer including anaplastic astrocytomas, glioblastomas (Hayatsu et al. 2008a) oesophageal and gastric adenocarcinoma (Laitinen et al. 2015; Borg et al. 2016), colorectal (Larsson et al. 2013; Larsson et al. 2016), breast (Sizemore et al. 2007; Snyder et al. 2015; Graves et al. 2016), hepatocellular (Flores-Tellez et al. 2015), pancreatic ductal (Heby et al. 2015; Saukkonen et al. 2015), oral squamous cell (Lin et al. 2014), urothelial bladder (Boman et al. 2013), ovarian (Ye et al. 2012), renal (Hsu et al. 2010), thyroid carcinoma (Yasuoka et al. 2008) and lymphoblastic and myeloid leukemia (Kelley et al. 2005; Riccioni et al. 2006; Nielsen and McNagny 2009). Podocalyxcin regulates cell adhesion and morphology and has a role in cancer development. Podocalyxcin increases the aggressiveness of tumours through induction of cell migration and invasion through interactions with the actin-binding protein EZR and the cytoskeleton, increasing outside-in cell signalling and MAPK and PI3K activity in cancer cells (Sizemore et al. 2007; Larrucea et al. 2008). Podocalyxcin has essential roles to play in neuritogenesis and synaptogenesis (Kiss and Rougon 1997; Eckhardt et al. 2000; Angata et al. 2007) and co-localises with synapsin and synaptophysin in synaptic vesicles (Vitureira et al. 2010). Synapsin tethers synaptic vesicles to cytoskeletal components regulating vesicle release into the synaptic gap during neural activation, synaptophysin co-ordinates neurotransmitter release from the synaptic vesicles (Thiel 1993; Kwon and Chapman 2011) when these fuse with the de-polarised post synaptic membrane (Cesca et al. 2010; Fornasiero et al. 2010; Bykhovskaia 2011; Song and Augustine 2015). Embryonic stem cells express podocalyxcin containing the R10G epitope thus are low sulfation isoforms of KS.

## Brain and cartilage aggrecan

**Fig. 5:**
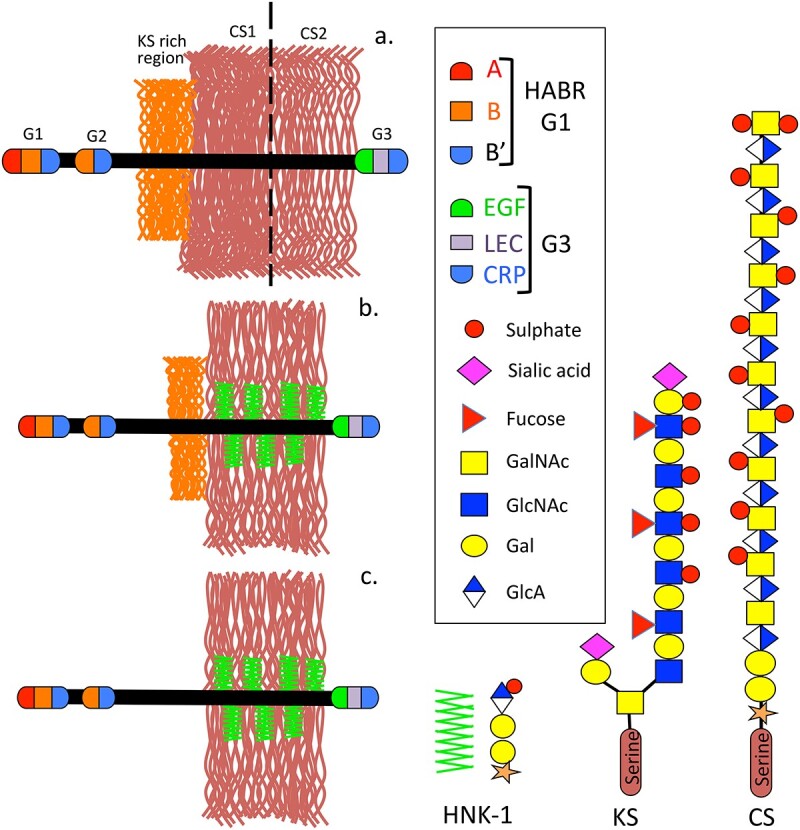
Schematic depiction of the structural organization of aggrecan showing the relative KS contents and HNK-1 epitope of articular cartilage (a), brain (b) and notochordal aggrecan (c). Image modified from (Hayes and Melrose 2020). Abbreviations used: A, immunoglobulin A HA binding sub-domain; B, B′ proteoglycan tandem repeat sub-domains; HABR, hyaluronan binding region; EGF, epidermal growth factor sub-domain; LEC, C-lectin subdomain; CRP, complement regulatory protein sub-domain; HNK-1, human natural killer cell carbohydrate epitope.

Aggrecan is a prominent component of perineuronal nets (PNNs) however brain aggrecan differs from cartilage aggrecan mainly in its KS content and the presence of HNK-1 trisaccharide (Hayes and Melrose 2020) (Fig. 5). Studies have shown that two out of every seven non-reducing termini of normal (Hardingham et al. 1994) and chondrosarcoma (Midura et al. 1995) aggrecan CS chains contain 4, 6-disulphated GalNAc. Non-reducing terminal GalNAc4S or GalNAc4,6S (CSE) can be linked to either a 4-sulphated or a 6-sulphated disaccharide. CS from juvenile and adolescent growth plate cartilage contains non-reducing terminal GalNAc4S, whereas in adult cartilages approximately half of the non-reducing termini are disulphated GalNAc4,6S (Plaas et al. 1998; West et al. 1999), representing an increase in aggrecan sulfation with tissue maturation. CS chains terminated in 4-sulphated GalNAc predominate in aggrecan from foetal to 15-year-old knee cartilage, whereas, in 22–72-year-olds, 50% of the CS chains are terminated in 4,6-disulphated GalNAc. GlcUA-4-sulphated GalNAc disaccharides terminate 7% of CS chains in foetal to 15-year-old cartilage but 3% in adults, GlcUA-6-sulphated GalNAc represents 9% of the CS chains in foetal to 72-year-old cartilage (Plaas et al. 1998). Non-reducing terminal 4,6-disulphated GalNAc residues are 60-fold more abundant than in interior regions of CS chains (Midura et al. 1995). C-6-S is predominantly distributed towards the non-reducing terminus and more abundant in mature cartilage to the detriment of C-4-S sulfation (Caterson 2012). While KS is present on brain aggrecan, its content is significantly reduced compared to cartilage aggrecan (Domowicz et al. 1995; Schwartz et al. 1996; Domowicz et al. 2003; Morawski et al. 2012). Notochordal aggrecan does not contain KS (Domowicz et al. 1995; Domowicz et al. 2000; Domowicz et al. 2003).

**Fig. 6:**
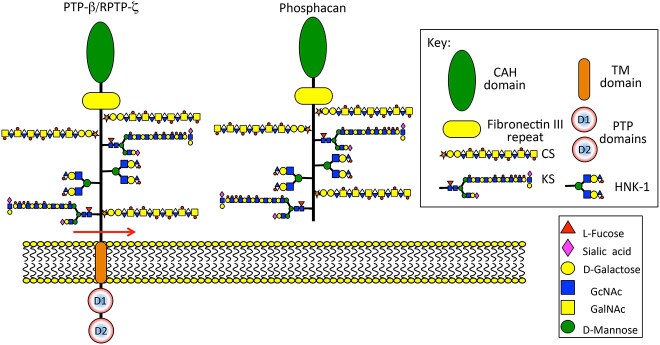
Schematic depictions of PTP-B/RPTP-Z and phosphacan showing their modular structures and attached glycosaminoglycan side chains including CS and KS side chains and the HNK-1 carbohydrate epitope. Figure modified from (Melrose 2018).

Human natural killer-1 (HNK-1) carbohydrate (HSO3-3GlcAβ1–3Galβ1-4GlcNAc-R) is highly expressed in the brain and is a component of brain aggrecan (Fig. 5b and c) and phosphacan (Fig. 6), prominent components of perineuronal nets with roles in cognitive learning, memory and neural plasticity (Celio 1993; Morise et al. 2014; Yabuno et al. 2015; Eill et al. 2020; Sinha et al. 2023). HNK-1 is a chain truncation signal thus brain aggrecan has a lower density of CS chains than cartilage aggrecan, HNK-1 however introduces additional functionalities to brain aggrecan. This is evident in the forms of aggrecan which direct cell migration and tissue morphogenesis during formation of neural networks (Hayes and Melrose 2020). The HNK-1 epitope is linked to phosphacan core protein through mannose residues (Morise et al. 2018).

## Phosphacan

Regulatory properties of KS-phosphacan with a specific sulfation pattern is responsible for the generation of long-term potentiation (LTP), a process involving persistent strengthening of synapses that leads to a long-lasting increase in signal transmission and may be related to cognitive learning and memory (Takeda-Uchimura et al. 2015; Hao et al. 2018; Eill et al. 2020). LTP is a specific form of activity-dependent synaptic plasticity and is a leading mechanism in learning and memory in mammals. Low sulfation KS isoforms of phosphacan/protein tyrosine phosphatase RPTPζ have important roles to play in PNN formation and function in cognitive learning. Time-dependent localization of KSPGs with different sulfation patterns in the song nuclei may underlie song learning in developing male zebra finches (Fujimoto et al. 2015; Melrose 2019a). Ubiquitous expression of BCD-4(+) low-sulfated KS was found in song centres in the brain of zebra song finches. These are regions in the brain with roles in cognitive song learning by the immature Zebrafinch from their elders. Song learning only occurs with immature males.

Synthesis of KS positive for the R-10G antibody in the adult brain is mediated by GlcNAc-6-sulfotransferase 3 (GlcNAc6ST3; encoded by *Chst5*) (Narentuya et al. 2019). Deficiency in GlcNAc6ST3 and GlcNAc6ST1, encoded by *Chst2*, completely abolishes KS biosynthesis. Protein-tyrosine phosphatase receptor type z1 (Ptprz1)/phosphacan in PNNs contain low sulfation KS. Extension of the KS is undertaken with GlcNAc by β1,3 *N*-acetylglucosaminyltransferase (Beta3Gn-T) (Takeda-Uchimura et al. 2022). R-10G + ve Protein tyrosine phosphatase receptor type z1 (Ptprz1, also known as PTPRZ, PTP-ζ, or RPTPβ)/phosphacan, is expressed in both developing and adult brains (Takeda-Uchimura et al. 2015; Narentuya et al. 2019). Two species of KS proteoglycans are present in brain tissue, (i) neuropil low sulphated R-10G + ve KS proteoglycan and (ii) high sulfation density microglial 5D4 + ve KS proteoglycan, although 5D4 positivity may occur in the low sulfation KS proteoglycan containing regions with tissue maturity or tissue injury.

## KS substituted proteins

KS can also occur on isolated proteins such as serotransferrin and thyroglobulin.

Serotransferrin is the fourth most abundant serum glycoprotein in humans. It consists of a 77 kDa polypeptide chain of 679 amino acids arranged in N-terminal and C-terminal domains with glycans attached to the C-terminus. Serotransferrin has two complex biantennary N-linked glycan chains terminated with sialic acids which are potential sites of KS substitution (Jamnongkan et al. 2019). KS substituted serotransferrin and thyroglobulin have been used as biomarkers of papillary thyroid carcinomas (Magro et al. 2003). Serotransferrin is also a stabilizing component interacting with actin microfilaments in KS electrosensory glycoconjugates in elasmobranch fish species (Zhang et al. 2018). Two forms of prostaglandin D synthase (PGD synthase) have been identified which are substituted with KS (Berryhill et al. 2001), a lipocalin and a hematopoietic enzyme (Urade and Eguchi 2002). Lipocalin-type PGD synthase is found in the CNS, male genital organs, human heart and as beta-trace, a major CSF protein. PGD synthase is lipophilic displaying high affinity binding to retinoids, thyroids, and bile pigments (Urade and Hayaishi 2000a; Urade and Hayaishi 2000b). Hematopoietic PGD synthase is found in peripheral tissues in antigen-presenting cells, mast cells, and megakaryocytes and is the first vertebrate homolog of α-glutathione S-transferase.

## Antibodies to the lactosamine regions of KS

Antibodies which detect non-branched and branched lactosamine structures in KS occur as autoantibodies (Feizi et al. 1971; Feizi et al. 1979; Feizi 1989; Young et al. 2007a). Commercial antibodies to Ii antigen have also now been developed (Hirvonen et al. 2013). The i antigen (linear poly-N-acetyllactosamine) from the Ii blood group system is a marker of umbilical cord blood (UCB) MSCs. Use of antibody phage technology to produce recombinant antibodies recognizing a structure from the surface of MSCs has facilitated construction of IgM phage display libraries from lymphocyte donors that displayed an elevated serum anti-i titer. Agglutination assays utilizing i antigen-positive red blood cells (RBCs) from UCB revealed six promising single-chain variable fragment (scFv) antibodies. Flow cytometry showed three of these antibodies recognized epitopes from the surface of UCB-MSCs. Further characterisation of these antibodies demonstrated they recognised a prominent cell surface i antigen on UCB-MSCs and RBCs (Hirvonen et al. 2013).

## A large number of KS antibodies testify to the structural diversity of KS

The large range of MAbs which have been developed to specific structural features of KS testify to the structural diversity of this molecule (Table 1).

**Table 1:** KS antibodies illustrate its structural complexity.

**Antibody clone**	**Epitope(s) identified**	**Ref**
TRA-1-60	Epitope identified is sensitive to neuraminidase, keratanase-I/II, and endo-β-D-galactosidase digestion. Epitopes identified are Galβ1-3GlcNAcβ1-3Galβ1-4GlcNAc and Galβ1-3GlcNAcβ1-3Galβ1-4GlcNAcβ1-6(Galβ1-3GlcNAcβ1-3)Galβ1-4Glc oligosaccharides expressed on podocalyxcin by pluripotent embryonic stem cells.	(Andrews et al. 1984; Badcock et al. 1999; Adewumi et al. 2007; Schopperle and DeWolf 2007; Natunen et al. 2011)
TRA-1-81	Epitope is resistant to neuraminidase but sensitive to endo-β-D-galactosidase, keratanase-I/II. Terminal Galβ1-3GlcNAcβ1-3Galβ1-4GlcNAc and Galβ1-3GlcNAcβ1-3Galβ1-4GlcNAcβ1-6(Galβ1-3GlcNAcβ1-3)Galβ1-4Glc epitope is expressed on cell surface podocalyxcin by pluripotent embryonic stem cells.	(Andrews et al. 1984; Badcock et al. 1999; Adewumi et al. 2007; Schopperle and DeWolf 2007; Natunen et al. 2011)
R-10G	Low sulfation poly *N*-acetyllactosamine KS epitope, resistant to digestion with peptide N-glycanase F, neuraminidase, fucosidase, chondrotinase ABC and heparinase, but completely susceptible to digestion with keratanase-I, keratanase-II and endo-β-galactosidase. Minimal epitope Galβ1-4GlcNAc(6S)β1-3Galβ1-4GlcNAc(6S)β1	(Magro et al. 2003; Nakano et al. 2012; Kawabe et al. 2013; Makanga et al. 2015; Nakao et al. 2023)
SSEA-1	Cell surface proteoglycan, glycoprotein and lipid glycan epitope produced by murine embryonic pluripotent stem cells.	(Ozawa et al. 1985)
4C4	Highly sulphated KS on embryonic tumour cell podocalyxcin.	(Fukuma et al. 2003)
5D4	di-sulphated hexasaccharide on oversulphated regions of KS.	(Caterson et al. 1983; Mehmet et al. 1986)
MZ15	Di-sulphated hepta and octa-saccharide KS oligosaccharides.	(Mehmet et al. 1986; Craig et al. 1987)
1B4	Monosulphated tetrasaccharide in KS	(Mehmet et al. 1986)
BCD-4	Peptide in KS rich region of aggrecan, also identified in brain song centres in Zebra Song finches, in cognitive song learning centres	(Glant et al. 1986; Fujimoto et al. 2015)
3D12/H7	Trisulphated fucosylated poly-*N*-acetyllactosamine in CS 1 and 2 regions of aggrecan.	(Fischer et al. 1996a)
D9B1	A sialo-KS epitope on endometrial KS-PGs.	(Smith et al. 1989; Hoadley et al. 1990; Aplin et al. 1998)
6D2/B5	Fucosyl-KS epitope.	(Baker et al. 1989)
R-6C	Sialylated KS. Siaα2-3Galβ1-3GlcNAc(6S)β1-3Galβ1-4GlcNAc(6S)β1	(Nakao et al. 2023)
R13E	Fucα1-2Galβ1-3GlcNAcβ1-3Galβ1	(Nakao et al. 2023)
R17F	lacto-N-fucopentaose. Fucα1-2Galβ1-3GlcNAβ1-3Galβ1-4Glc	(Nakao et al. 2023)
SV2	High sulfation KS chains on SV2 PG of synaptic vesicles.	(Scranton et al. 1993; Sinouris et al. 2009)
EFG-11	Tri KS disaccharides.	(Papageorgakopoulou et al. 2002)
1/14/16H9	Specific equine KS antibody.	(Okumura and Fujinaga 1998; Okumura et al. 2000)
BKS-1(+)	Keratanase generated D-GlcNAc 6-sulphate KS stub neo-epitope.	(Akhtar et al. 2008)

## The biodiversity of KSPG form and function

KSPGs are widely distributed in a diverse range of tissues (Table 2). Aggrecan is a lectican large KS and CS PG with space filling and water imbibing properties through interactions with HA and the formation of massive macro-aggregate link protein stabilized ternary structures. Aggrecan is widely distributed in articular, hyaline, elastic and fibrocartilages, costal, nasal and tracheal cartilages, larynx, outer ear and epiglottis. Aggrecan equips tissues with weight bearing properties and mechanically supports elastic and collagen fibres in elastic and tensional tissues to provide tissue deformability and resilience. Aggrecan is heavily substituted with ~100 CS and 20 KS chains representing ~90% of the mass of this PG. KS is localised in a KS-rich region adjacent to the CS rich region and G1, G2 and interglobular domains (IGDs). Aggrecan in notochordal tissue contains less KS than the form of aggrecan present in articular cartilage. Aggrecan is a major lectican found in perineural nets found in brain tissues and has proposed roles in cognitive learning, synaptic plasticity and memory.

**Table 2:** The diversity of KS proteoglycans, glycoproteins, glycoconjugates and isolated KS substituted proteins.

**Protein**	**Distribution**	**Functions**	**Reference**
**KS-proteoglycans of tensional and weight-bearing connective tissues**			
Aggrecan	Large PG of cartilage, CNS, tendon, IVD	Tissue hydration, weight bearing. Inhibits neurite outgrowth, provides axon repulsive guidance cues	(Fryer et al. 1992; Kiani et al. 2002)
Fibromodulin	Widely distributed in cornea, cartilage, tendon, IVD, meniscus	Regulates collagen fibrillogenesis and inflammatory cytokines/growth factors, cell proliferation and cell signaling.	(Chakravarti 2002; Schaefer and Iozzo 2008)
Keratocan	Widespread distribution in collagenous tissues	Bears 38% homology to lumican and shares many of its functional properties	(Corpuz et al. 1996; Conrad and Conrad 2003; Igwe et al. 2011)
Lumican	Widespread distribution in collagenous tissues	Multifunctional properties with growth factors, cytokines, morphogens. Marker of tissue pathology. Roles in collagen fibrillogenesis determines optical clarity of the cornea. Roles in inflammation and tissue repair. Lumican peptides have MMP-inhibitor activity, C-terminal 13C lumikine peptide has growth factor activity. Lumican peptides have anti-tumor activity.	(Chakravarti et al. 1998; Chakravarti et al. 2000; Brezillon et al. 2009a; Brezillon et al. 2009b; Carlson et al. 2010; Chen et al. 2010; Hassell and Birk 2010; Nikitovic et al. 2011; Brezillon et al. 2013; Coulson-Thomas et al. 2013; Liu et al. 2013; Pietraszek et al. 2013; Pietraszek et al. 2014; Gesteira et al. 2017)
Osteoadherin (osteomodulin)	Cartilage/bone growth plate interface	Cell binding bone KSPG, may regulate mineralization	(Sommarin et al. 1998)
Mimecan (osteoglycin)	Broad distribution in connective tissues	Corneal mimecan is sulfated but it is not sulfated in other tissues. Has roles in bone induction	(Funderburgh et al. 1997; Corpuz et al. 2000)
CD44	Epidermal/CNS KS-CD44 isoform	Ubiquitous HA receptor occurring as alternatively spliced forms substituted with KS, CS or HS	(Takahashi et al. 1996)
Bone sialoprotein-II (BSP-II)	80 kDa core protein has sialic acid and *N*- and *O*-linked KS chains	BSP-II is a KSPG in compact rabbit bone, BSP-II from other species does not contain KS. Related KSPG identified in rat calvaria and in medullary bone in laying birds	(Masubuchi et al. 1975; Kinne and Fisher 1987; Ganss et al. 1999; Nakamura et al. 2001; Hadley et al. 2016)
**KS-proteoglycans of mucinous tissues**			
MUC1	epithelial distribution	transmembrane epithelial KSPG, heavily O-glycosylated, sialylated forms 200–500 nm layer on cell surface	(Aplin et al. 1998; Brayman et al. 2004)
Mucous KSPG	220 kDa 5D4 + ve KSPG	KSPG of cervical mucous secretions	(Fischer et al. 1996a; Fischer et al. 1996b; Fischer et al. 2001)
Podocalyxcin	240 kDa R10G + ve	Mucin-like, sialomucin cell surface KS–PG related to CD34. Anti-adhesive. Widespread epithelial distribution	(Vitureira et al. 2005; Nielsen and McNagny 2009; Vitureira et al. 2010)
Zona pellucida protein-3 (PZP-3)	zona pellucida	An N-linked polylactosamine sulfated KS protein with oocyte–sperm receptor interactive activity	(Noguchi and Nakano 1992; Nakano et al. 1996)
Keratinocyte perlecan	Epidermis	Hybrid KS-HS-CS basement membrane proteoglycan with roles in ECM stabilization and growth factor binding	(Knox et al. 2005)
KS glyco-conjugate Ampoulles of Lorenzini	Ultra sensitive electrosensory gel in elasmobranch fish used in electrolocation	KS glycoconjugate stabilised by actin microfibrils, serotransferrin disulphide stabilised interactions with as mucin-like glycoprotein containing low sulfation KS chains	(Josberger et al. 2016; Zhang et al. 2018)
**KS-proteoglycans of the PNS/CNS**			
Synapse vesicle protein 2 (SV2)	12 span membrane KSPG, 100/250 kDa forms and 3 isoforms SV2A, B, C	Storage/neurotransmitter transport in synaptic vesicles/neuroendocrine cells. KS of SV2 interactive component of a smart gel delivery system	(Bajjalieh et al. 1992; Feany et al. 1992; Bajjalieh et al. 1993; Scranton et al. 1993; Bajjalieh et al. 1994)
Phosphacan, RPTP-β/PTPζ,	PNS/CNS has KS, CS and HNK-1 substitution. Phosphacan is the ecto-domain of PTPζ	PTPζ is a type I transmembrane glycoprotein, carbonic anhydrase motif interacts with pleiotrophin and midkine to promote neurite outgrowth activity	(Garwood et al. 1999; Garwood et al. 2003; Faissner et al. 2006; Morise et al. 2014)
**Miscellaneous KS-substituted proteins**			
Transferrin, thyroglobulin	Associated with papillary thyroid carcinoma	KS epitope is capped with α2-3 *N*-acetylneuraminic acid	(Magro et al. 2003)
Prostaglandin-D synthase	28 kDa KS-glycoprotein produced by bovine corneal keratocytes	Corneal retinoid transporter, also found in seminal plasma, rat brain and spinal cord, rat cochlea, human prostate, human and rat epididymis and testes	(Berryhill et al. 2001)

CNS, central nervous system; SV2, synaptic vesicle protein 2; RPTPβ, receptor protein tyrosine phosphatase β; PTP ζ, protein tyrosine phosphatase ζ.

Several members of the SLRP family (fibromodulin, lumican, keratocan, mimecan) are substituted with a few small low sulfation *N*-linked KS chains. The SLRPs regulate collagen fibrillogenesis, interact with cytokines and growth factors and regulate cell proliferation, cell signaling, matrix assembly and tissue repair. Some SLRP members (PRELP, mimecan, osteoadherin) have minimally sulphated KS chains and roles in the laying down of bone and anchorage of basement membrane to adjacent connective tissue. Bone sialoprotein-II from compact rabbit bone has small low sulphated KS chains. KS-glycoproteins have been identified in epithelial tissues. MUC1 is a widely distributed mucin glycoprotein which contains small low-sulfation KS chains. Podocalyxcin, a 240 kDa sialylated mucin like cell membrane proteoglycan of embryonic stem cells contains low sulfation KS chains however in adult tissues and in tumours its KS chains are highly sulphated. SLRPs associated with corneal development have been shown to display variable high and low sulfation KS chains in a spatiotemporal manner apparently related to the diameter of collagen fibrils in specific tissue regions. After the cornea the brain is a rich source of KS in the human body and these decorate a number of brain PGs. Phosphacan is one of the most abundant KSPGs in brain tissue and has roles in the regulation of neuronal development and repair processes and perineuronal net formation. Synaptic protein-2 (SV2) is a 12 span neurotransmitter transport and storage proteoglycan which contains 3 large highly sulphated KS chains. Endometrial KSPG and PZP-3 have roles in fertilization and implantation. Variants of CD44 and perlecan have also been described bearing KS chains. KS chains have also been detected on transferrin and thyroglobulin in papillary thyroid carcinoma where the KS chains are of diagnostic value. Prostaglandin D synthase has roles in neurophysiological functions, hormone release, and pain responses. Keratocytes synthesise 28 kDa Prostaglandin D synthase, a known retinoid transporter as a KS-proteoglycan (Berryhill et al. 2001).

## Corneal proteoglycan diversity

The vertebrate cornea is a highly specialized transparent tissue that covers the anterior surface of the eye, three main tissue layers are discernable, (i) the outer stratified squamous epithelium, (ii) the intermediate stroma, and (iii) the inner endothelium. A number of KS-SLRP family members (lumican, keratocan, mimecan) have critical roles to play in the laying down of regularly organized orthogonal thin collagen fibrils of uniform size to ensure optical clarity in the cornea (Chakravarti et al. 1998; Corpuz et al. 2000; Dunlevy et al. 2000; Chakravarti et al. 2003; Chen et al. 2010; Chen et al. 2015) (Table 3). Fibromodulin promotes the assembly of larger collagen fibers in the limbus and sclera providing mechanical support to the periphery of the cornea ensuring the tension throughout the cornea is uniform (Chakravarti et al. 2003; Chen et al. 2010). Gene targeting of specific SLRP family members confirms their critical roles in collagen fibrillogenesis and the 3D ECM organization in the cornea with several ocular diseases apparent when they are dysfunctional or deleted (Funderburgh et al. 1989; Edward et al. 1990; Chakravarti et al. 2000; Pellegata et al. 2000; Kao and Liu 2002; Tasheva et al. 2002; Liu et al. 2003; Ho et al. 2014). Sulfation is a key functional determinant in KS and is not uniform throughout the cornea. A comparison of high charge density 5-D-4 + ve KS and moderately sulfated 1-B-4 + ve KS shows that the former is uniformly distributed across the cornea, but 1-B-4 reactivity is highest in the peripheral regions and lowest in the central cornea (Ho et al. 2014). KS-SLRPs have widespread distributions in corneal and retinal tissues and have critical roles to play in ECM organization in these tissues. A thin film of high charge density 5-D-4 + ve KS has also been localized in the surface epithelium of the cornea which may be due to a KS substituted mucin-like glycoprotein which provides boundary lubrication against shear stresses, protection from microbial infection and aids in the hydration of the cornea (Hodges and Dartt 2013; Baudouin et al. 2018; Hampel et al. 2018).

**Table 3:** Matrix KSPGs detected in connective tissues of the eye.

Tissue	Proteoglycan	Reference
Cornea		
Epithelium	Lumican, keratocan, mucin 5D4 KS	(Saika et al. 2002; Ho et al. 2014)
Sclera	fibromodulin, PRELP, aggrecan, keratocan, mimecan	(Pellegata et al. 2000; Tanihara et al. 2002; Tasheva et al. 2002; Liu et al. 2003; Chen et al. 2014; Chen et al. 2015; Massoudi et al. 2016)
Limbus	fibromodulin, lumican, PRELP	(Tanihara et al. 2002)
Stroma	lumican, keratocan, mimecan	(Pellegata et al. 2000; Tanihara et al. 2002; Tasheva et al. 2002; Liu et al. 2003; Chen et al. 2014; Chen et al. 2015; Massoudi et al. 2016)
Endothelium	Lumican	(Tanihara et al. 2002)
Retina		
Choroid	aggrecan, fibromodulin, lumican, mimecan	(Inatani and Tanihara 2002)
Pigmented epithelium	fibromodulin, PRELP, aggrecan,	(Inatani and Tanihara 2002)
Rod and Cone Interphotoreceptor pericellular matrix	aggrecan, PRELP, fibromodulin, lumican, mimecan, keratocan, high molecular weight mucin	(Plantner 1992; Inatani and Tanihara 2002)
Photoreceptor segment	aggrecan, fibromodulin, lumican, mimecan	(Inatani and Tanihara 2002)
Ganglion cell layer	aggrecan, PRELP, fibromodulin, phosphacan	(Inatani et al. 2000; Tanihara et al. 2002; Popp et al. 2003; Popp et al. 2004)
Nerve rich layer	phosphacan, aggrecan	(Inatani and Tanihara 2002; Popp et al. 2003; Popp et al. 2004; Tanihara et al. 2002)
Optic nerve	aggrecan, phosphacan	(McAdams and McLoon 1995; Inatani et al. 2000; Popp et al. 2003; Popp et al. 2004)

The cornea KS is not highly sulphated during early embryonic development however by E10 the chick cornea displays significant deposition of a matrix containing highly sulphated KS and this regulates the spatial organization of collagen fibril bundles extruded by the embryonic corneal cells (Gealy et al. 2007; Eghrari et al. 2015). Electron microscopy shows highly-sulfated KS closely associated with bundles of regularly arranged collagen fibrils. A steady increase in 5D4 + ve proteoglycan in the cornea then occurs over E12-18 (Young et al. 2007b) and with time collagen-KS associations are widespread through the epithelial, stromal and endothelial layers with multiple KS-SLRP members directing collagen fibrillogenesis and 3D ECM organization (Quantock and Young 2008; Chen et al. 2014; Chen et al. 2015).

High-sulfation KS defines the regular spatial organization of collagen fibrils in newly synthesized collagen fibre bundles extruded into the extracellular environment. The accumulation of highly sulfated KS in the E12-E18 chick finely tunes local matrix organization and collagen fibril spacing during corneal growth.

Furthermore, keratocan has neuro-directory properties, and growth factor and morphogen interactivities supportive of tissue developmental processes (Gealy et al. 2007; Conrad et al. 2010; Weyers et al. 2013). The KS chains of keratocan have interactive properties with a range of neuroregulatory proteins which direct nerve migration. Keratocan mRNA is developmentally regulated in the anterior–posterior and dorsal ventral axes during early (E2-E3) chick embryonic development (Conrad and Conrad 2003). In the developmental cornea, accumulation of highly sulphated PGs in the posterior stroma inhibits nerve penetration however keratocan in the anterior epithelium permits nerve penetration (Schwend et al. 2012a). Trigeminal nerve growth in the embryonic chick reaches the corneal margins by E5, and is initially repelled by highly sulphated ECM PGs, it subsequently encircles the corneal margins over E5-E8 until entering the cornea on E9. KS mediated Robo-Slit cell signaling guides trigeminal nerve development and migration during this innervative period (Schwend et al. 2012b). The interactive properties of KS with these nerve regulatory proteins exemplifies its neurogenic and neurodirective properties (Conrad et al. 2010).

## KS facilitates electrolocation, an ultrasensitive sensory modality

**Fig. 7:**
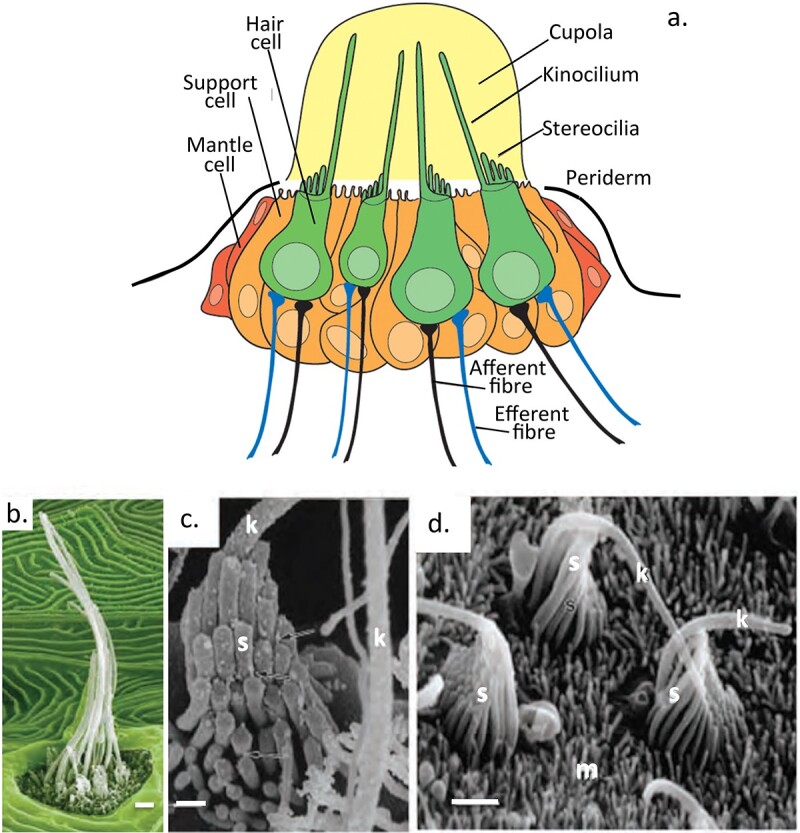
A schematic diagram of the zebrafish lateral line neuromast and surrounding sensory hair and support cells showing the stereocilia and kinocelium of the hair cells which detect signals from the aqueous environment where information on water displacement is transduced into signals which provide information on spatial awareness used in electrolocation (a). Electron micrographs showing stereocilia with cupola removed (pseudocoloured) (b) and of stereocilia hair bundles (s), kinocilium (k) and microvilli (m) in the gummy shark (c, d). Image a. reproduced from (Chiu et al. 2008). Images b-d reproduced from (Gardiner and Atema 2014). The detection of sound transmission in the cochlea involves the transduction of acoustic information into electrical signals by the sensory hair cells. Highly sensitive imaging methodologies have been developed to examine the structure and function of the stereocilia in sound perception. These include 3D electron tomography density mapping and second harmonic imaging microscopy (Gueta et al. 2007; Sazzed et al. 2018). Scanning electron microscopy has also been used to analyse the development of stereocilia and the structure and composition of hair cell stereociliary bundles (Ivanchenko et al. 2021; Mishra et al. 2022).

**Fig. 8:**
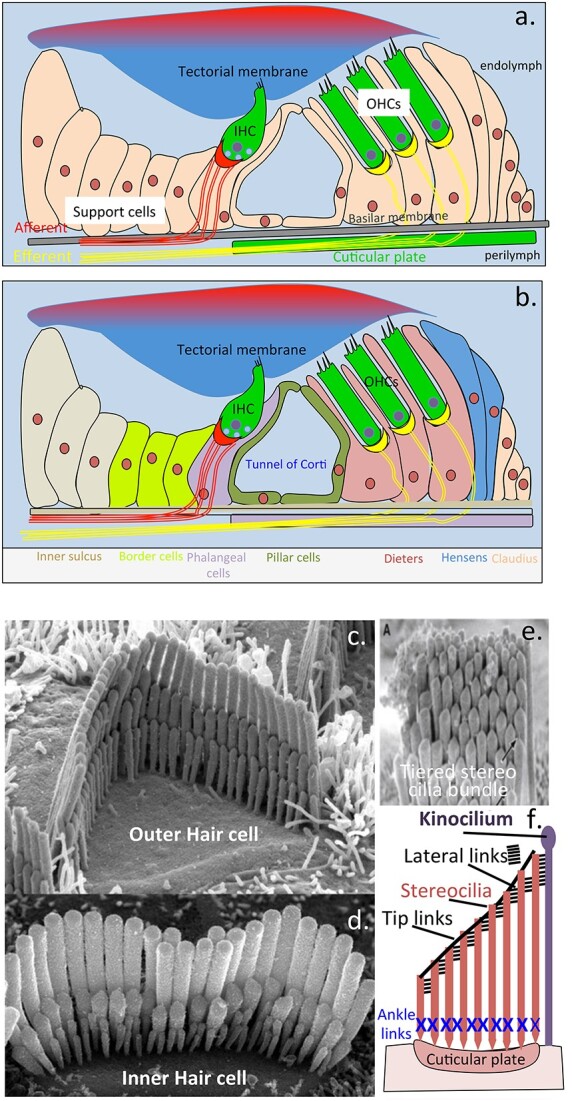
Schematic depiction of the tectorial membrane and inner and outer hair cells with their hierarchical stereocilia and their attachments responsible for detection of sound waves that stimulate the tectorial membrane (a). The adjacent support cells are also shown (b). Electron micrographs depict the hierarchical rows of stereocilia on the outer (c) and inner hair cells (d) and in a stereocilia bundle (e). Schematic depiction of a stereocilia bundle showing the interconnections between them and the adjacent kinocilium (f). Images c-e reproduced under licence attribution-NonCommercial 4.0 international (CC BY-NC 4.0) hair cells of inner ear by Dr David Furness welcome collection.

In fish, the sensory hair cells of the lateral line are directly exposed to the water surrounding the fish (Fig. 7). Stereocilia detect directional changes in fluid flow to provide spatial awareness, ultrasensitive KS-sensory gels in the ampullae of lorenzini which detect proton gradients generated by the muscular activity of prey fish species by a process known as electrolocation also contribute to environmental sensory processes. While the process of electrolocation as a sensory system has not been retained by terrestrial animals there are nevertheless obvious parallels in the mammalian cochlea which is also an organ immersed in a sound conductive medium where acoustic signals are transmitted by the tectorial membrane to stereocilia of the outer then inner sensory hair cells, these contain tectorin-α, a KS mucin like glycoprotein on the stereocilia membrane surface. This KS-glycoconjugate may serve a similar role to the electrosensory gels of elasmobranch fish. It is noteworthy that both of these polymers contain low sulfation KS chains, however high charge density KS (5D4) is also found on the stereocilia tip where it may have some electro-sensory properties relating to its charge status which however await detailed characterization (Fig. 8). High density sulphated KS is an electroconductive anionic Ca2+ interactive molecule and thus may function in some way with the electromechanical pores found in the tip of stereocilia. Hair-cell transduction is astonishingly sensitive, the mechanotransduction machinery of hair cells responds to minute physical displacements on a submillisecond timescale (Gillespie and Müller 2009; Fettiplace and Kim 2014). These transduction channels are cation selective showing a preference for Ca^2+^, in proteoglycans Ca2+ can act as a counterion to the KS side chains of PGs which may possibly act as a local Ca2+ reservoir. While several components of the sensory mechanotransduction machinery in stereocilia have been identified it is still unclear how these pores function (Qiu and Müller 2018). The high charge KS chains in this region of the stereocilia may be yet another molecule worthy of further evaluation. KS is an ultrasensitive proton detection molecule (Selberg et al. 2019). Proton (H^+^) conductivity is important in many natural phenomena in proton voltage-gated ion channels (DeCoursey 2003). Stereocilia that decorate sensory hair cells in the cochlea have important roles to play in the detection of acoustic signals and transduction of this information through nerve networks to the brain for signal interpretation. Stereocilia are hierarchically organized in rows on the sensory hair cells, these are interconnected to one another by linkages at the tip along the shaft and at the base of the stereocilia facilitating anchorage to the cuticular plate (Fig. 8c–f). These interconnections have essential roles to play in the perception of sound. Stereocilia also have mechanotransductive channels in their tips with roles in the electromechanical transduction of displacements in the stereocilia transmitted by acoustic signals which impact the tectorial membrane in the cochlea resulting in movement of K+ into the stereocilia from the endolymph of the tectorial membrane. This also results in an influx of Ca2+ into the stereocilia through voltage gated ion channels and movement of synapatic vesicles containing neurotransmitters to the hair cell interface with a neural network. Fusion of the vesicle with the hair cell membrane results in release of neurotransmitters carried in the vesicles to communicating neural networks which interface with the sensory hair cells. The outer hair cells contain a further 12 span membrane motor protein, prestin which acts an amplifying system which allows a >100 fold in sound detected by the inner hair cells.

The entire inner ear of the cochlea is bathed in a cushioning fluid, called the endolymph. Sound waves impacting on the gelatinous tectorial membrane results in the exudation of endolymphatic fluid which is transferred to the stereocilia of the inner and outer sensory hair cells (IHCs and OHCs) in the cochlea. The opening of mechanically gated ion channels at the tips of the stereo-cilia facilitates the entry of endoymph into the IHCs and OHCs. Stereocilia are arranged in three hierarchical rows, cross-linked by several types of extracellular links (Cartagena-Rivera et al. 2019) coupling the mechanical properties of stereocilia bundles. Stereocilin connects outer hair cell stereocilia to one another and to the tectorial membrane (Verpy et al. 2011; Avan et al. 2019). In the mammalian cochlea, mechanoelectrical transducer (MET) channels located at the tips of the middle and shorter rows of the stereociliary hair bundle are responsible for passage of K+ from the endolymph of the tectorial membrane into IHCs and OHCs. This initiates an influx of Ca2+ into the sensory hair cells through voltage gated ion channels which results in mobilisation of intracellular vesicles containing neurotransmitters to the synaptic interface with neural networks and transfer of neurotransmitters resulting in neural transduction. A number of highly specialized proteins are responsible for stereocilia assembly and function during acoustic stimulation and these also provide mechanical stiffening and anchorage to the cuticular plate. Tightly packed parallel arrangements of actin microfilaments provide mechanical support to the stereo-cilia (Park and Bird 2023) however the upper region of the stereocilia is stabilized by interactions between the cytoplasmic tail of cadherin 23 and its actin anchoring protein harmonin forming large protein aggregates attached to the cytoskeleton providing mechanical support and control of the gating properties of the MET channels in the tip of the stereocilia (Wu et al. 2012). Interactions between Cadherin 23, myosin VIIa and harmonin form a stabilizing ternary complex, interaction with membrane phospholipids and motor proteins such as prestin control the gating properties of the MET channels (Bahloul et al. 2010). Prestin is a plasma membrane protein that has been proposed to act as a cochlear amplifier (Dallos et al. 2006; Yu et al. 2006; Mellado Lagarde et al. 2008; Raphael 2022). Specific proteins have also been identified with precise roles in stereocilia anchorage to the cuticular plate and interactions with actin microfilaments which control the precise alignment and orientation of the stereocilia (Sekerková et al. 2006; Pollock et al. 2016; Katsuno et al. 2019; Michel et al. 2020). Specific proteins also regulate actin cytoskeletal dynamics and the biogenesis of stereocilia (Johnson et al. 2012; Goodman and Zallocchi 2017). The mechanoelectrical transduction channels in the tip of stereocilia are key functional regions of the sensory hair cells (Fettiplace and Kim 2014; Cunningham and Müller 2019). Membrane electromechanics are important in the auditory functions of the stereocilia (Araya and Brownell 2016) and synaptic transmission in the cochlea (Fettiplace 2017).

The tectorial membrane is a gel-like, acellular connective tissue overlying the organ of Corti auditory sensory structure. The tectorial membrane is essential to the synchronous sound deflection properties of the stereocilia of the IHC and OHCs and central to auditory transduction (Richardson et al. 2008; Lukashkin et al. 2010; Sellon et al. 2019). Collagen, primarily type II, is the major protein of the tectorial membrane, smaller amounts of type IX collagen is also present. Information on the tectorial membrane proteoglycans however is incomplete.

The tectorial membrane has an important role to play in the mechanism of how the cochlea transduces mechanical energy (acoustic sound waves) transmitted through the cochlea into neural excitation (the process of hearing) by the IHCs and OHCs (Richardson et al. 2008; Lukashkin et al. 2010; Goodyear and Richardson 2018; Sellon et al. 2019). The stereocilia tips of the sensory hair cells of the cochlea are embedded in the tectorial membrane (Hakizimana and Fridberger 2021) and associated with their mechanical properties (Gueta et al. 2006). Distortions in the apical surface of the tectorial membrane by acoustic sound waves results in a release of endolymph rich in K+ ions which enter the stereocilia through gated pores in its tip and this sets in motion a series of events that lead to acoustic signals eliciting a neural response in the neural network associated with the OHCs and IHCs. Examination of the tectorial membrane by electron microscopy has identified a number of layers to the apical surface with parallel type II collagen fibres arranged perpendicular to the apical surface. Type IX collagen is also present and has important roles to play in the hearing process (Asamura et al. 2005; Suzuki et al. 2005). Type IX KO mice have impaired hearing (Suzuki et al. 2005). Stickler syndrome is a connective tissue disorder characterized by ocular, skeletal, orofacial and auditory dysfunction (Acke and De Leenheer 2022). The most common form of Stickler syndrome (*COL2A1*) is characterized by mild to high hearing loss due to deficiency in Col2A1, Col9A1, Col9A2, Col9A3 gene expression. In articular cartilage, a tissue rich in type II collagen, type IX collagen is layed down on the surface of type II collagen fibres and determines the type II collagen fibre size and 3D organization (Müller-Glauser et al. 1986; Eyre 2002; Eyre et al. 2006). Type IX collagen is a proteoglycan and contains a single CS chain (Brewton et al. 1991).

Type II collagen in the apical surface of the tectorial membrane is cross-linked by a KS-glycoprotein, tectorin (Tsuprun and Santi 1997; Andrade et al. 2016). This surface region of the tectorial membrane contains a high sulfation form of KS detected by MAb 5D4 (Thalmann et al. 1993). A further 5D4 + ve KS proteoglycan in cornea, lumican, also controls collagen fibrillogenesis and 3D organization and is critical to corneal optical clarity (Kao and Liu 2002; Chen et al. 2014). Tectorin however is not a 5D4 + ve proteoglycan but contains low sulfation KS chains (Munyer and Schulte 1994; Swartz and Santi 1997). Further work needs to be undertaken to identify this surface region KS-proteoglycan in the tectorial membrane. A uronic acid containing chondroitin-4 and chondroitin-6-sulphate proteoglycan has also been detected in extracts of tectorial membrane, this may be type IX collagen but further confirmatory work needs to be undertaken to verify this possibility (Munyer and Schulte 1994; Munyer and Schulte 1995; Swartz and Santi 1997).

**Fig. 9:**
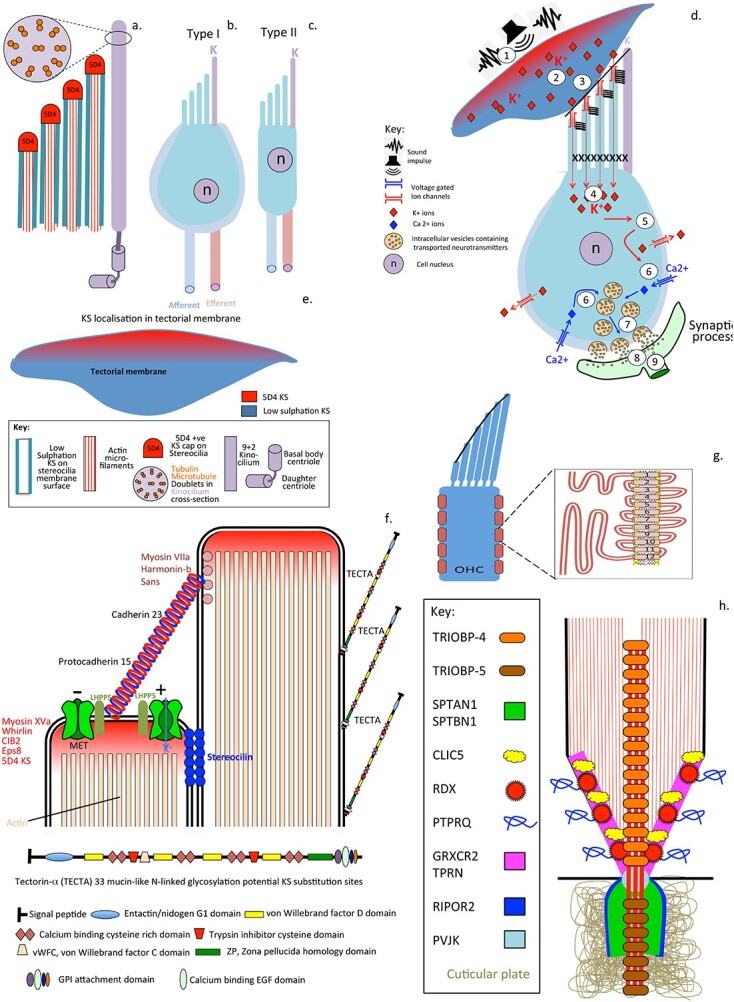
Structural organization of the stereocilia root of cochlear sensory hair cells and adjacent kinocilium showing its internal 9 + 2 microtubular structure and 5D4 KS at the stereocilia tips, internal bundles of supportive actin microfibrils and low sulfation KS on the stereocilia shaft (a), inner (b) and outer hair cells (c). Sequential changes (1–9) in the sensory hair cells when sound waves impact the tectorial membrane resulting in movement of K+ from endolymph through pores at the stereocilia tip resulting in an influx of Ca2+. Mobilization of vesicles, membrane fusion and release of neurotransmitters (d). The apical surface of the tectorial membrane is 5D4 + ve, internal regions contain a low sulfation KS isoform (e). Organisation of two adjacent stereo cilia tips, anchoring cadherin 23/protocadherin 15, myosin VIa, Harmonin-b, scaffolding protein sans at the anchor point and myosin XVa, Whirlin, CIB2 (calcium and integrin-binding protein 2), protein tyrosine kinase Eps8 (epidermal growth factor receptor pathway substrate 8) on an adjacent stereocilium. Stereocilin also anchors the stereocilia. The tip of the sterocilia contains 5D4 + ve KS, the shaft of stereocilia contains tectorin-α (f). Prestin is a 12 span motor protein sound amplifier for inner hair cells (g). Actin interactive scaffolding proteins have specific anchorage roles in the root of the hair stereocilium. TRIOBP-4 and 5 (TRIO and F-actin binding protein) are actin bundling proteins, Sptan1 is a filamentous cytoskeletal protein, SPTB1 (spectrin beta chain, brain 1) inhibits inflammatory responses, CLIC5 (chloride intracellular channel protein 5) associates with actin-based cytoskeletal structures, radixin (RDX) is an actin binding cytoskeletal protein. PTPRQ (protein tyrosine phosphatase receptor type Q) and myosin VI maintain stereocilia organization, GRXCR2 (glutaredoxin domain-containing cysteine-rich protein-2) and TPRN (taperin) have roles in stereocilia morphogenesis. RIPR2 (rho family-interacting cell polarization regulator 2) regulates myoblast and hair cell differentiation. PVJK (Pejvakin) regulates functional properties of auditory pathway neurons (h). Figure (h) modified from (Pacentine et al. 2020).

Tectorin, KS-proteoglycan is associated with the cochlea tectorial membrane and has audiosensory properties. Tectorin cDNA predicts a high molecular weight protein of 239,034 Da with 33 potential N-glycosylation sites, and the smaller beta-tectorin is a 36,074 Da protein with 4 consensus N-glycosylation sites (Legan et al. 1997). The high molecular weight form of tectorin is present in the tectorial membrane of the cochlea of the inner ear, stereo hair bundles and olfactory mucus layer (Killick and Richardson 1997). Tectorin has a buoyant density in CsCl density gradient ultracentrifugation typical of a glycoprotein rather than a proteoglycan. Tectorin however is sensitive to keratanase and endoglycosidase digestion but does not react with MAb 5-D-4 (Killick et al. 1995). The surface regions of the tectorial membrane however stain with MAb 5-D-4 as do the tips of the stereo cilia sensory hairs that are displaced by auditory signals transmitted by the tectorial membrane (Fig. 9). The deeper layers of the tectorial membrane and the outer membraneous regions of the stereo cilia shafts contain tectorin with low sulfation KS chains. Ion-transfer to the sensory hair stacks and audio-transductive signalling to attached neural networks forms the basis of hearing. Tectorin can be selectively labeled with radiosulphate, its lack of 5-D-4 reactivity suggests it is a KS-proteoglycan containing low charge density forms of KS (Killick et al. 1995). α-Tectorin is heavily N-glycosylated and β-tectorin contains at least 4 sites which may be substituted by KS. The 5-D-4 positive staining on the surface of the tectorial membrane suggests the presence of a further KS-proteoglycan containing high charge density KS or an iso-form of tectorin containing high charge density KS chains. Specific mutations in the α-tectorin gene (TECTA) have been shown to lead to specific forms of deafness (Verhoeven et al. 1998; Balciuniene et al. 1999). As to why the tips of electrosensory stereocilia have high charge density KS in their tips that interact with the tectorial membrane while the shaft of the stereocilia contain low sulphated KS isoforms is not known. It is possible that the electrorepulsive properties of high charge density KS may be too disruptive of the cohesivity of sterocilia hair bundles which are interconnected by a number of specific protein crosslinks as already discussed. Maintenance of these stereocilia interconnections is important in the provision of the rigidity of hair bundles which is required to maintain their acoustic signaling properties. Disruption of these stereocilia protein networks that interconnect adjacent stereocilia results in deafness. Furthermore, KS is a highly interactive GAG, FGF2 and sonic hedgehog bind strongly to KS and these may have roles in the biogenesis and maintenance of stereocilia (Weyers et al. 2013).

## Anchorage points at stereocilia tips are of functional importance in accoustic signaling

The tips of adjacent stereo cilia are attached to one another by a cadherin 23/protocadherin 15 strand using Myosin VIa, Harmonin-b, scaffolding protein Sans as an anchor point on one stereocilium and Myosin XVa, Whirlin CIB2 (Calcium and integrin-binding protein 2), protein tyrosine kinase Eps8 (Epidermal growth factor receptor pathway substrate 8) on the adjacent stereocilium (Fig. 9f). Stereocilin also anchors the stereocilia together at a separate point. The tip of the sterocilia also contains 5D4 + ve KS whose functional role is not known, this surrounds MET electromechanical pores in the tip of the stereocilia. The membrane along the shaft of stereocilia contains tectorin-α, α low sulfation KS glycoprotein. Prestin is a 12 span transmembrane motor protein found in outer hair cells which has been proposed to act as a sound amplifier for inner hair cells increasing the detection of acoustic signals by 100 fold (Fig. 9g). Polycystic Kidney and Hepatic Disease 1-Like 1 (PKHD1L1), a large single span transmembrane extracellular protein of 4,249 amino acids is also a component of the surface coat of stereocilia and is required for normal hearing in mice (Wu et al. 2019).

## Stabilisation of the base of stereocilia and their anchorages into the cuticular plate

A number of interactive proteins have been identified which provide stabilizing features to the base of stereocilia of importance in the transductive properties of stereocilia and acoustic signal transmission (Fig. 9h). TRIOBP isoforms 4 and 5 (TRIOBP-4/-5, TRIO and F-actin Binding Proteins) are actin-bundling stereocilia stabilizing proteins, loss of these proteins is associated with hearing loss demonstrating the essential roles they play in stereocilia structure and function (Bao et al. 2015). Multicolor total internal reflection fluorescence microscopy shows how decoration of actin filaments by Tropomyosin (TPM) isoforms influences actin assembly (Jansen and Goode 2019). SPTAN1, nonerythroid spectrin αII. Sptan1 KO mice exhibit rapid deafness, abnormal stereocilia and cuticular plates. These findings illustrate SPTAN1 as a critical molecule for HC stereocilia morphology and auditory function (Yao et al. 2022). CLIC5, Chloride intracellular channel 5 protein complexes with MYO6 forming stabilizing linkages between the plasma membrane and actin cytoskeleton at the base of stereocilia (Salles et al. 2014). Ezrin/radixin/moesin (ERM) proteins cross-link and integrate actin filaments with plasma membranes (Kitajiri et al. 2004). Glutaredoxin domain-containing cysteine-rich protein 2 (GRXCR2) is localized at the base of stereocilia and is necessary for stereocilia morphogenesis and auditory perception (Liu et al. 2015; Li et al. 2021). RIPOR2 (RHO family interacting cell polarization regulator 2) is a membrane protein with roles in myotube formation and in regulation of cell adhesion, polarization, and migration involved in stereocilia morphogenesis and is essential to the hearing process (Diaz-Horta et al. 2018).TPRN (Taperin), together with its binding proteins CLIC5 and PTPRQ, form concentric rings in the taper region of stereocilia which have important stabilizing and functional roles (Qi et al. 2024). PJVK, Pejvakin binds to and colocalizes with the rootlet component TRIOBP at the base of stereocilia. Hair cells of pejvakin-deficient mice develop normal rootlets, but hair bundle morphology and mechanotransduction are affected before the onset of hearing indicating Pevjakin has roles to play in the functional maturation of stereocilia (Kazmierczak et al. 2017). TRIOBP isoforms 4 and 5 (TRIOBP-4/-5) are actin-bundling proteins associated with roles in stereocilia stabilization (Bao et al. 2015).

## Concluding remarks

Keratan sulfate is a truly remarkable glycosaminoglycan and much still needs to be learned of its properties. This review has attempted to outline some of these properties and to show why KS is of particular importance in neurosensory processes. Further work needs to be conducted on the KS-proteoglycans associated with the tectorial membrane and stereocilia of sensory hair cells. The ability to detect proteoglycans and glycoconjugates containing low sulfation isoforms of KS is opening up yet another fascinating aspect of the biology of KS which needs to be expanded. The electrical and proton interactive properties of KS suggest it has properties that may be of application in potential futuristic areas of nanoelectronics, and in the development of artificial synapses, memristors and molecular switches in nanomachines. Such developments are eagerly anticipated as is their application in neural pathobiology and repair biology.

## Data Availability

All data is available from the publications listed in the bibliography.

## CRediT author statement

James Melrose (Conceptualization [lead], Data curation [lead], Formal analysis [lead], Funding acquisition [lead], Project administration [lead], Writing—original draft [lead], Writing—review & editing [lead].
